# Randomized, double-blind, placebo-controlled phase III study of ixazomib plus lenalidomide-dexamethasone in patients with relapsed/refractory multiple myeloma: China Continuation study

**DOI:** 10.1186/s13045-017-0501-4

**Published:** 2017-07-06

**Authors:** Jian Hou, Jie Jin, Yan Xu, Depei Wu, Xiaoyan Ke, Daobin Zhou, Jin Lu, Xin Du, Xiequn Chen, Junmin Li, Jing Liu, Neeraj Gupta, Michael J. Hanley, Hongmei Li, Zhaowei Hua, Bingxia Wang, Xiaoquan Zhang, Hui Wang, Helgi van de Velde, Paul G. Richardson, Philippe Moreau

**Affiliations:** 1 Department of Hematology, Chang Zheng Hospital, The Second Military Medical University, Shanghai, 200003 China; 2 First Hospital Affiliated Zhe Jiang Medical University, Hangzhou, China; 3 Institute of Hematology & Blood Diseases Hospital, Chinese Academy of Medical Science & Peking Union Medical College, Tianjin, China; 4 The First Affiliated Hospital of Soochow University, Jiangsu, China; 5 The Third Affiliated Hospital of Beijing Medical University, Beijing, China; 6 Peking Union Medical College Hospital, Beijing, China; 7 Peking University People’s Hospital, Beijing, China; 8 Guangdong General Hospital/Guangdong Academy of Medical Sciences, Guangzhou, China; 9 Xijing Hospital, The Fourth Military Medical University, Xi’an, China; 10 Ruijin Hospital, Shanghai Jiao Tong University, Shanghai, China; 11 The Third Hospital Xiang Ya Medical University, Changsha, China; 12 Millennium Pharmaceuticals, Inc. (a wholly owned subsidiary of Takeda Pharmaceutical Company Limited.), Cambridge, MA USA; 13 Dana-Farber Cancer Institute, Boston, MA USA; 14 University Hospital Hôtel-Dieu, Nantes, France

**Keywords:** China, Ixazomib, Multiple myeloma, Oral, Overall survival, Progression-free survival, Proteasome inhibitor, Relapsed/refractory

## Abstract

**Background:**

The China Continuation study was a separate regional expansion of the global, double-blind, placebo-controlled, randomized phase III TOURMALINE-MM1 study of ixazomib plus lenalidomide–dexamethasone (Rd) in patients with relapsed/refractory multiple myeloma (RRMM) following one to three prior therapies.

**Methods:**

Patients were randomized (1:1) to receive ixazomib 4.0 mg or placebo on days 1, 8, and 15, plus lenalidomide 25 mg on days 1–21 and dexamethasone 40 mg on days 1, 8, 15, and 22, in 28-day cycles. Randomization was stratified according to number of prior therapies, disease stage, and prior proteasome inhibitor exposure. The primary endpoint was progression-free survival (PFS). In total, 115 Chinese patients were randomized (57 ixazomib-Rd, 58 placebo-Rd).

**Results:**

At the preplanned final analysis for PFS, after median PFS follow-up of 7.4 and 6.9 months, respectively, PFS was improved with ixazomib-Rd versus placebo-Rd (median 6.7 vs 4.0 months; HR 0.598; *p* = 0.035). At the preplanned final analysis of overall survival (OS), after median follow-up of 20.2 and 19.1 months, respectively, OS was improved with ixazomib-Rd versus placebo-Rd (median 25.8 vs 15.8 months; HR 0.419; *p* = 0.001). On the ixazomib-Rd and placebo-Rd arms, respectively, 38 (67%) and 43 (74%) patients reported grade ≥3 adverse events (AEs), 19 (33%) and 18 (31%) reported serious AEs, and 4 (7%) and 5 (9%) died on-study. The most frequent grade 3/4 AEs were thrombocytopenia (18%/7% vs 14%/5%), neutropenia (19%/5% vs 19%/2%), and anemia (12%/0 vs 26%/2%).

**Conclusions:**

This study demonstrated that PFS and OS were significantly improved with ixazomib-Rd versus placebo-Rd, with limited additional toxicity, in patients with RRMM.

**Trial registration:**

ClinicalTrials.gov, NCT01564537

**Electronic supplementary material:**

The online version of this article (doi:10.1186/s13045-017-0501-4) contains supplementary material, which is available to authorized users.

## Background

Multiple myeloma (MM) accounts for approximately 13% of hematologic cancers [1]. In the USA and European Union, the age-standardized annual incidence is approximately 4.5 to 7 cases per 100,000 population [2, 3]; MM is currently less common in Asian countries, but the incidence is increasing [4, 5]. In China, the age-standardized annual incidence of MM in 2005 was estimated to be 0.6 per 100,000 [6], and the age-standardized mortality rate has been estimated as 0.6 per 100,000 deaths in 2013 [5]. A large retrospective analysis of outcomes of Chinese patients indicated that, at diagnosis, patients have more advanced-stage disease, renal dysfunction, and bone destruction compared with Western patients [7].

The introduction of novel therapies such as proteasome inhibitors and immunomodulatory drugs has significantly improved survival in MM over the past two decades [8]. Outcomes are improving further through the treatment paradigm of long-term or continuous therapy [9, 10]. However, despite these improvements, MM remains incurable. New treatment options are needed for patients with relapsed/refractory MM (RRMM), and specific investigation of these therapies in Asian populations is important in the context of differences in the clinical profile of MM and treatment patterns compared with in North America and Europe [4].

Ixazomib is the first oral proteasome inhibitor to enter the clinic. It is approved by the US Food and Drug Administration and the European Medicines Agency for use, in combination with lenalidomide and dexamethasone (Rd), for the treatment of patients with MM who have received at least one prior therapy [11, 12]. These decisions were based on the global, double-blind, placebo-controlled, randomized phase III TOURMALINE-MM1 study of ixazomib-Rd versus placebo-Rd in 722 patients with RRMM following one to three prior lines of therapy [13]. Ixazomib-Rd demonstrated a significant 35% improvement in progression-free survival (PFS) versus placebo-Rd (median 20.6 vs 14.7 months; hazard ratio (HR) 0.74), with limited additional toxicity. As overall survival (OS) data were not mature at the primary analysis, the global study continued in a double-blind, placebo-controlled fashion [13].

The global TOURMALINE-MM1 study included only 6 Chinese patients. Thus, a separate regional expansion study—the China Continuation study—was conducted, following the approval of Rd for RRMM by the China State Food and Drug Administration in 2013 [14], with the aims of evaluating ixazomib-Rd versus placebo-Rd in a Chinese patient population and of expanding the worldwide information on the addition of ixazomib to Rd in the treatment of RRMM. The China Continuation study had a similar study design to the global study and, as an intended registration study, was executed with the same stringency [13].

## Methods

### Study design and participants

The China Continuation study was a randomized, double-blind, placebo-controlled study conducted at 11 centers in China; patients were enrolled between May 8, 2014, and May 8, 2015. The study was a separate regional expansion of the global phase III TOURMALINE-MM1 study [13] and had identical eligibility criteria (see Additional file 1), the same treatment schema and assessment methodology (with the exception of cytogenetics assessment), and the same primary and secondary endpoints (with the exception of outcomes in patients with high-risk cytogenetics, and quality-of-life and healthcare resource utilization endpoints, as described below). Briefly, patients aged ≥18 years with a confirmed diagnosis of MM, measurable disease, and creatinine clearance ≥30 mL/min, who had relapsed and/or refractory disease having received one to three prior treatments, were eligible. All patients provided written informed consent. The protocol was approved by ethics committees/review boards at all participating centers; the study was conducted in accordance with the International Conference on Harmonization Good Clinical Practice guidelines.

### Assessments

Patients were randomized (1:1) to receive ixazomib-Rd or placebo-Rd, stratified by number of prior therapies (1 vs 2 or 3), International Staging System (ISS) disease stage [15] (I or II vs III), and prior proteasome inhibitor exposure (yes vs no). Patients, investigators, treating physicians, and all study personnel were blinded to assigned treatment. Patients received ixazomib 4.0 mg capsules or matching placebo on days 1, 8, and 15, plus lenalidomide 25 mg, days 1–21, and dexamethasone 40 mg, days 1, 8, 15, and 22, in 28-day cycles. Lenalidomide dose was 10 mg in patients with creatinine clearance <60 mL/min (per lenalidomide label). Treatment was continued until disease progression or unacceptable toxicity.

Patients were assessed every 28 days for response and disease progression using central laboratory data on M-protein and FLC levels. Blinded independent review committee (IRC) evaluation of response/progression was done per International Myeloma Working Group 2011 response criteria [16]. Patients who came off study treatment were followed every 4 weeks for PFS and every 12 weeks for OS. Adverse events (AEs) were evaluated using the National Cancer Institute’s Common Terminology Criteria for AEs v4.03. Unlike the global study, quality-of-life and healthcare resource utilization data were not collected in the China Continuation study.

The pharmacokinetics of ixazomib were characterized in a subset of Chinese patients who were randomized to ixazomib-Rd and consented to intensive pharmacokinetic sampling. Methods for pharmacokinetics analyses are summarized in Additional file 1.

### Statistical analysis

The primary endpoint was PFS by blinded IRC assessment; the IRC was the same as the one constituted for the global study. Secondary endpoints included OS, time to progression (TTP), response rates, duration of response (DOR), safety, and pharmacokinetics. Unlike the global study, efficacy endpoints in subgroups based on cytogenetic risk were not assessed because the central laboratory used in the global study did not have a representative in China with validated tests for cytogenetics, and, per local export regulations, biologic samples could not be exported in real time for central laboratory cytogenetics testing.

The sample size of 115 patients was intended to fulfill Chinese regulatory requirements with the goal of evaluating consistency with the global study in the treatment effect of ixazomib-Rd versus placebo-Rd. With HR = 0.728, the probability of observing HR <0.9 is estimated to be ~80% with ~60 PFS events. Further details on events required for the preplanned final analyses of PFS and OS are summarized in Additional file 1.

SAS Version 9.1 or higher was used for all statistical analyses. A two-sided, unstratified log-rank test was used to compare the treatment groups with respect to PFS, TTP, and OS. In addition, an unadjusted unstratified Cox model was used to estimate the HR and 95% confidence intervals (CIs) for the treatment effect, overall, and in patient subgroups. Kaplan–Meier survival curves and medians were determined for each treatment group and subgroup. Response rates were compared between the treatment groups using an unstratified Cochran–Mantel–Haenszel test. Other efficacy endpoints were summarized descriptively.

## Results

### Patients

**Fig. 1:**
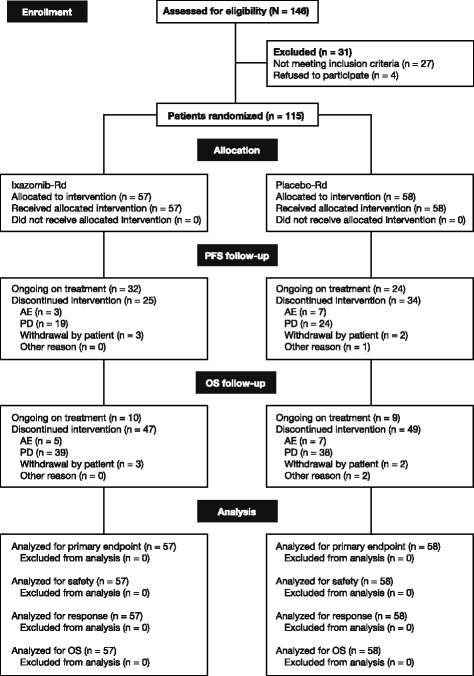
CONSORT diagram of patient disposition and flow through the study

A total of 115 patients were randomized to receive ixazomib-Rd (*n* = 57) or placebo-Rd (*n* = 58); at data cut-off for the final analysis for OS, 47 (82%) and 49 (84%) patients in the ixazomib-Rd and placebo-Rd arms, respectively, had discontinued treatment (Fig. 1). Patient demographics and baseline disease characteristics were generally well balanced between treatment arms (Table 1).

**Table 1:** Baseline demographics and disease characteristics of patients enrolled in the C16010 China Continuation Study

Baseline characteristics	Ixazomib-Rd (*n* = 57)	Placebo-Rd (*n* = 58)	Overall (*n* = 115)
Median age, years (range)	61.0 (30–76)	61.5 (36–80)	61.0 (30–80)
Patient age, *n* (%)			
≤65 years	42 (74)	41 (71)	83 (72)
>65–75 years	14 (25)	14 (24)	28 (24)
>75 years	1 (2)	3 (5)	4 (3)
Male sex, *n* (%)	41 (72)	38 (66)	79 (69)
Baseline ECOG performance status, *n* (%)			
0	25 (44)	26 (45)	51 (44)
1	31 (54)	29 (50)	60 (52)
2	1 (2)	3 (5)	4 (3)
MM subtype at study entry, *n* (%)			
IgG	29 (51)	31 (53)	60 (52)
IgA	11 (19)	14 (24)	25 (22)
Light chain only	13 (23)	8 (14)	21 (18)
Other	4 (7)	5 (9)	9 (8)
ISS stage at initial diagnosis, *n* (%)			
I	11 (19)	11 (19)	22 (19)
II	17 (30)	14 (24)	31 (27)
III	21 (37)	21 (36)	42 (37)
Unknown	8 (14)	12 (21)	20 (17)
ISS stage at study entry, *n* (%)			
I	31 (54)	38 (66)	69 (60)
II	21 (37)	16 (28)	37 (32)
III	5 (9)	4 (7)	9 (8)
Creatinine clearance, mL/min, *n* (%)			
<30	0	1 (2)	1 (<1)
30–<60	4 (7)	8 (14)	12 (10)
60–<90	28 (49)	23 (40)	51 (44)
≥90	25 (44)	26 (45)	51 (44)
Median time since initial MM diagnosis, months (range)	29.5 (3–143)	28.6 (1–141)	28.7 (1–143)
Lines of prior therapy, *n* (%)			
1	25 (44)	26 (45)	51 (44)
2	20 (35)	24 (41)	44 (38)
3	12 (21)	8 (14)	20 (17)
Disease status at study entry, *n* (%)			
Relapsed^a^	15 (26)	13 (22)	28 (24)
Refractory^b^	28 (49)	33 (57)	61 (53)
Relapsed and refractory^c^	14 (25)	12 (21)	26 (23)
Prior therapy exposure, *n* (%)			
Prior proteasome inhibitor (all bortezomib)	34 (60)	36 (62)	70 (61)
Prior immunomodulatory drug therapy	52 (91)	47 (81)	99 (86)
Lenalidomide	3 (5)	7 (12)	10 (9)
Thalidomide	52 (91)	45 (78)	97 (84)
Thalidomide-refractory	37 (65)	35 (60)	72 (63)
Prior corticosteroids	57 (100)	58 (100)	115 (100)
Dexamethasone	56 (98)	57 (98)	113 (98)
Prednisone	17 (30)	20 (34)	37 (32)
Prior melphalan	24 (42)	24 (41)	48 (42)
Prior stem cell transplant	8 (14)	12 (21)	20 (17)

*Abbreviations*: *ECOG* Eastern Cooperative Oncology Group, *ISS* International Staging System, *MM* multiple myeloma

^a^Patients who had relapsed from at least one previous treatment but were not refractory to any previous treatment

^b^Patients who were refractory to at least one previous treatment but were not relapsed to any previous treatment

^c^Patients who were relapsed from at least one previous treatment and additionally were refractory to at least one previous treatment. Refractory disease was defined as disease progression on treatment or progression within 60 days after the last dose of a given therapy

### Efficacy

**Fig. 2:**
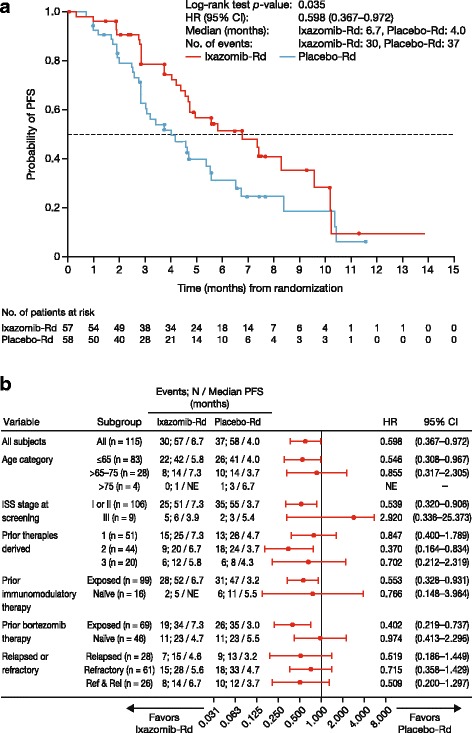
PFS (time from randomization to first documentation of PD or death) with ixazomib-Rd and placebo-Rd at data cut-off for primary and final analysis of PFS (median follow-up for PFS of 7.4 and 6.9 months, respectively). **a** Kaplan–Meier analysis of PFS by IRC assessment in the intent-to-treat population. **b** Forest plot of PFS in prespecified patient subgroups

At data cut-off for the primary and final analysis of PFS, the median follow-up for PFS was 7.4 months in the ixazomib-Rd arm and 6.9 months in the placebo-Rd arm. Per IRC assessment, 67 PFS events (confirmed progression or death) had occurred in 30 (53%) and 37 (64%) patients in the ixazomib-Rd and placebo-Rd arms, respectively. There was a significant 67% improvement in PFS with ixazomib-Rd versus placebo-Rd (HR 0.598, 95% confidence interval (CI) 0.367–0.972; *p* = 0.035, log-rank test); median PFS was 6.7 months (95% CI 4.63–9.53) versus 4.0 months (95% CI 2.79–5.52) (Fig. 2a). A PFS benefit with ixazomib-Rd versus placebo-Rd was seen across prespecified subgroups defined by age and prior therapy exposure (Fig. 2b).

The overall response rate (ORR) was 56 versus 31% with ixazomib-Rd versus placebo-Rd (odds ratio (OR) 2.84 [95% CI 1.33–6.10]; *p* = 0.007), including rates of very good partial response or better of 25 versus 12% (Table 2); among responding patients, median DOR was 7.4 versus 5.6 months. A significant benefit in favor of ixazomib-Rd was observed for TTP. At data cut-off for the final analysis of PFS, 28 (49%) and 35 (60%) patients in the ixazomib-Rd and placebo-Rd arms, respectively, had documented progression. There was a 71.5% improvement in TTP with ixazomib-Rd versus placebo-Rd (HR 0.583; 95% CI 0.353–0.963; *p* = 0.032); median TTP was 7.3 months (95% CI 4.70–9.53) versus 4.1 months (95% CI 2.99–5.52).

**Table 2:** Summary of best confirmed response to treatment with ixazomib-Rd and placebo-Rd

Best confirmed response, *n* (%)	Ixazomib-Rd (*n* = 57)	Placebo-Rd (*n* = 58)	*p* value (unstratified Cochran–Mantel–Haenszel test)
ORR (≥PR) (95% CI)	32 (56) (42–69)	18 (31) (20–45)	0.007
≥VGPR rate (95% CI)	14 (25) (14–38)	7 (12) (5–23)	0.084
CR	3 (5)	0	0.078
PR	29 (51)	18 (31)	–
VGPR	11 (19)	7 (12)	–
SD	17 (30)	17 (29)	–
PD	6 (11)	15 (26)	–
Not evaluable	2 (4)	8 (14)	–
	*n* = 32	*n* = 18	
Time to response, median (IQR)	1.0 (0.9–1.8)	1.0 (0.9–1.9)	–
DOR, median (95% CI)	7.4 months (6.21–NE)	5.6 months (2.73–9.46)	–
Responders who had not progressed at data cut-off, *n* (%)	19 (59)	7 (39)	

Time to response: time from first documentation of PR or better to first documentation of PD; Duration of response: time from first documentation of partial response or better to first documentation of progression

*Abbreviations*: *CR* complete response, *DOR* duration of response, *NE* not estimable, *ORR* overall response rate, *PD* progressive disease, *PR* partial response, *SD* stable disease, *VGPR* very good partial response

Reversal of renal insufficiency, defined as an increase in creatinine clearance from <50 mL/min at baseline to >60 mL/min post-baseline, was reported in 2 of 2 patients on the ixazomib-Rd arm and 0 of 5 patients on the placebo-Rd arm who had creatinine clearance <50 mL/min at baseline.

**Fig. 3:**
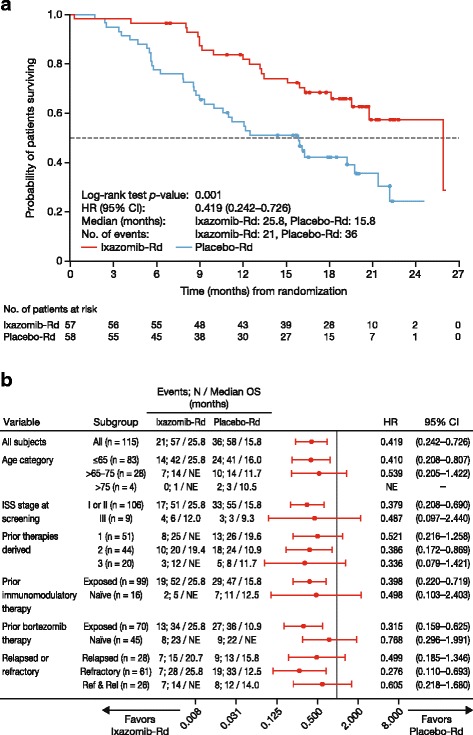
OS with ixazomib-Rd and placebo-Rd at data cut-off for final analysis of OS (median follow-up of 20.2 and 19.1 months, respectively). **a** Kaplan–Meier analysis of OS in the intent-to-treat population. **b** Forest plot of OS in prespecified patient subgroups

At the final analysis of PFS, OS data were not mature, and the study remained blinded. At data cut-off for the subsequent final analysis for OS, only 1 patient had been unblinded; median follow-up for OS was 20.2 and 19.1 months in the ixazomib-Rd and placebo-Rd arms, respectively. Twenty-one (37%) and 36 (62%) patients had died in the ixazomib-Rd and placebo-Rd arms, respectively, primarily due to myeloma (16/21 [76%] and 27/36 [75%] patients, respectively). There was a significant 139% improvement in OS with ixazomib-Rd versus placebo-Rd (HR 0.419; 95% CI 0.242–0.726; *p* = 0.001), and a 10-month difference between arms in median OS, which was 25.8 months (95% CI 19.42—not estimable) versus 15.8 months (95% CI 9.95–21.29) (Fig. 3a). Additional sensitivity analyses of OS, investigating potential confounding effects of subsequent therapies, showed consistent results (see Additional file 2: Table S1). An OS benefit with ixazomib-Rd versus placebo-Rd was seen across prespecified subgroups defined by age, disease status, and prior therapy exposure (Fig. 3b). Among patients in the ixazomib-Rd and placebo-Rd arms with creatinine clearance ≥60 mL/min, the OS HR was 0.455 (95% CI 0.256–0.809; *p* = 0.006), and median OS was 25.8 versus 16.0 months.

At data cut-off for the final analysis of OS, 30 (53%) and 25 patients (43%) in the ixazomib-Rd and placebo-Rd arms, respectively, had received subsequent therapy (Additional file 2: Table S2). In these 55 patients, 15 of 30 patients (50%) in the ixazomib-Rd group and 18 of 25 patients (72%) in the placebo-Rd group had died; in ad hoc analyses, in these 55 patients, there was a 108% improvement in OS (HR 0.48; *p* = 0.0355); median OS was 20.7 versus 15.8 months. In 60 patients who had not received subsequent therapy, 6 of 27 patients (22%) in the ixazomib-Rd group and 18 of 33 patients (55%) in the placebo-Rd group had died; in these 60 patients, there was a 208% improvement in OS (HR 0.324; *p* = 0.0122); median OS was not reached versus 15.8 months.

The type of subsequent therapy was well balanced between treatment groups (Additional file 2: Table S2), except for higher rates of alkylator-based and “other” subsequent therapies in the ixazomib-Rd arm. An OS benefit in favor of ixazomib-Rd was observed in patients who received subsequent alkylator therapy (24 and 14 patients in the ixazomib-Rd and placebo-Rd arms, respectively; HR 0.582; 95% CI 0.240–1.410; *p* = 0.225), in patients who received subsequent therapies without alkylators (6 and 11 patients; HR 0.502; 95% CI 0.135–1.861; *p* = 0.293), and in patients who did not receive subsequent therapies with alkylators (33 and 44 patients who received no subsequent therapy or subsequent therapies without alkylators; HR 0.350; 95% CI 0.164–0.746; *p* = 0.004). In 15 and 11 patients in the ixazomib-Rd and placebo-Rd arms who received subsequent bortezomib, median OS from start of subsequent bortezomib was 11.8 versus 8.0 months (HR 0.675; 95% CI 0.253–1.803; *p* = 0.430).

Based on the OS findings, following unblinding of the study, patients in the placebo-Rd arm are being given the option to cross over and receive ixazomib-Rd.

### Treatment exposure and safety

At the data cut-off for the final analysis of OS, patients in the ixazomib-Rd and placebo-Rd arms had received a median of 9.0 (range 1–25) and 6.5 (range 1–25) treatment cycles, respectively (Table 3), with the longer duration of therapy with ixazomib-Rd reflecting the improved PFS in the ixazomib-Rd arm in this treat-to-progression study design. Median treatment duration in the ixazomib-Rd and placebo-Rd arms was 272 and 181 days, respectively. The median relative dose intensity (RDI) for ixazomib and placebo was 100%, and in the ixazomib-Rd and placebo-Rd arms, respectively, the median RDI for lenalidomide was 97.1 and 99.8%, and for dexamethasone was 97.5 and 98.2%. Table 3 reports dose reductions required due to AEs.

**Table 3:** Summary of treatment exposure and overall safety profile of ixazomib-Rd and placebo-Rd

Variable	Ixazomib-Rd (*n* = 57)	Placebo-Rd (*n* = 58)
Median number of cycles, *n* (range)	9 (1–25)	6.5 (1–25)
Patients receiving ≥10 cycles, *n* (%)	28 (49)	20 (34)
Median treatment duration, days (range)	272 (8–679)	181 (16–712)
Relative dose intensity, %, mean (standard deviation)/median (range)^a^		
Ixazomib or placebo	96.0 (8.13)/100 (67–100)	98.7 (2.95)/100 (89–100)
Lenalidomide	89.7 (15.96)/97.1 (38–100)	94.5 (14.87)/99.8 (53–137)
Dexamethasone	91.7 (13.52)/97.5 (50–100)	95.2 (9.80)/98.2 (45–100)
Rates of AEs, *n* (%)		
Any AE	57 (100)	57 (98)
Any drug-related AE	54 (95)	57 (98)
Any grade ≥3 AE	38 (67)	43 (74)
Any drug-related grade ≥3 AE	33 (58)	37 (64)
Any serious AE (SAE)	19 (33)	18 (31)
Any drug-related SAE	11 (19)	7 (12)
AEs resulting in dose reduction of any study drug	12 (21)	11 (19)
Ixazomib/placebo^b^	1 (2)	0
Lenalidomide^c^	7 (12)	9 (16)
Dexamethasone^d^	5 (9)	3 (5)
AEs resulting in discontinuation of any study drug^e^	8 (14)	8 (14)
AEs resulting in discontinuation of study regimen	5 (9)	6 (10)
On-study deaths^f^	4 (7)	5 (9)

^a^Relative dose intensity defined as total amount of dose taken divided by total prescribed dose across treated cycles, as a percentage

^b^Ixazomib dose reduction required due to peripheral neuropathy and herpes zoster

^c^Lenalidomide dose reductions on the ixazomib-Rd arm associated with AEs of leukopenia, neutropenia, fatigue, pneumonia, glomerular filtration rate decreased, platelet count decreased, peripheral neuropathy, and acute kidney injury (dose reduction associated with >1 AE in some patients). Lenalidomide dose reductions on the placebo-Rd arm associated with AEs of granulocytopenia, pancytopenia, glomerular filtration rate decreased, neutrophil count decreased, musculoskeletal pain, acute kidney injury, chronic kidney disease, renal failure, and pruritic rash. One patient on each arm required two lenalidomide dose reductions

^d^Dexamethasone dose reductions on the ixazomib-Rd arm associated with AEs of diarrhea, face edema, peripheral edema, bronchitis, herpes zoster, lung infection, diabetes mellitus, peripheral neuropathy, and chronic kidney disease (dose reduction associated with >1 AE in some patients). One patient on the ixazomib-Rd arm required two dexamethasone dose reductions. Dexamethasone dose reductions on the placebo-Rd arm associated with AEs of pancytopenia, cataract, asthenia, and malaise (dose reduction associated with >1 AE in some patients)

^e^Only osteolysis (3 patients on the ixazomib-Rd arm), pneumonia (2 patients on the ixazomib-Rd arm), and lung infection (2 patients on the placebo-Rd arm) were reported in >1 patient

^f^Defined as deaths during treatment or within 30 days after the last dose of any study drug

The overall safety profile is summarized in Table 3; 67 and 74% of patients in the ixazomib-Rd and placebo-Rd arms, respectively, reported at least one grade ≥3 AE and 33 and 31% reported at least one serious AE (SAE). Common AEs plus other AEs of clinical importance are summarized in Table 4. Reflecting numerically lower rates of anemia with ixazomib-Rd compared to placebo-Rd (35 vs 53%; grade 3/4 12%/0 vs 26%/2%), red blood cell transfusions were required in 6 (11%) versus 12 (21%) patients and human erythropoietin was received by 2 (4%) and 3 (5%) patients in the ixazomib-Rd and placebo-Rd arms, respectively. Rates of gastrointestinal events were numerically higher with ixazomib-Rd compared with placebo-Rd. All events were grade 1/2 severity except for one case of grade 3 diarrhea in the ixazomib-Rd arm, and the majority of events occurred within the first 3 months of treatment. The rate of liver-related AEs was also numerically higher with ixazomib-Rd (21 vs 9%), primarily due to a higher rate of grade 1/2 increases in alanine aminotransferase (12 vs 5%). A higher rate of herpes zoster reactivation was observed in the ixazomib arm (21 vs 3%). Among patients who were not receiving antiviral prophylaxis (46 and 50 patients on the ixazomib-Rd and placebo-Rd arms, respectively), the rates were 24 and 2%, whereas in 11 and 8 patients who were receiving antiviral prophylaxis, the respective rates were 9 and 13%. No patients discontinued treatment due to herpes zoster. The rate of rash was 18 versus 21%, and the rate of peripheral neuropathy was 7 versus 10% with ixazomib-Rd versus placebo-Rd, with no grade ≥3 events reported in either arm. Rates of cardiovascular AEs were ≤5% in both arms. Events of hypercalcemia or elevated calcium were reported in 1 (2%) and 6 (10%) patients in the ixazomib-Rd and placebo-Rd arms, respectively; renal failure was reported in 0 and 2 (3%) patients. No thromboembolic AEs were reported in either arm; 98% of patients received thromboprophylaxis while receiving lenalidomide. There were no new primary malignancies reported in either arm.

**Table 4:** Common AEs reported in ≥10% of the safety population in either the ixazomib-Rd or placebo-Rd arm, plus other AEs of clinical importance

AE	Ixazomib-Rd (*n* = 57)			Placebo-Rd (*n* = 58)		
	All grades	Grade 3	Grade 4	All grades	Grade 3	Grade 4
Common AEs, *n* (%)						
Thrombocytopenia^a^	39 (68)	10 (18)	4 (7)	36 (62)	8 (14)	3 (5)
Neutropenia^b^	28 (49)	11 (19)	3 (5)	29 (50)	11 (19)	1 (2)
Anemia^c^	20 (35)	7 (12)	0	31 (53)	15 (26)	1 (2)
Pneumonia^d^	20 (35)	10 (18)	1 (2)	15 (26)	10 (17)	0
Upper respiratory tract infection	19 (33)	3 (5)	0	14 (24)	1 (2)	0
Leukopenia	17 (30)	5 (9)	0	10 (17)	1 (2)	0
Hepatotoxicity^e^	12 (21)	3 (5)	0	5 (9)	0	0
Herpes zoster	12 (21)	4 (7)	0	2 (3)	0	0
Weight decreased	11 (19)	0	0	9 (16)	0	0
Diarrhea	10 (18)	1 (2)	0	4 (7)	0	0
Rash^f^	10 (18)	0	0	12 (21)	0	0
Cough	9 (16)	0	0	3 (5)	0	0
Pyrexia	7 (12)	0	0	8 (14)	0	0
Hypokalemia	7 (12)	3 (5)	1 (2)	2 (3)	0	0
Bone pain	6 (11)	1 (2)	0	4 (7)	1 (2)	0
Insomnia	6 (11)	0	0	6 (10)	0	0
Lymphopenia	6 (11)	2 (4)	0	1 (2)	0	0
Fatigue	5 (9)	1 (2)	0	7 (12)	0	0
Hypoesthesia	4 (7)	0	0	7 (12)	0	0
Hyperglycemia	2 (4)	0	0	6 (10)	1 (2)	0
Other AEs of clinical interest, *n* (%)						
Other gastrointestinal AEs						
Nausea	5 (9)	0	0	2 (3)	0	0
Vomiting	5 (9)	0	0	2 (3)	0	0
Peripheral neuropathies^g^	4 (7)	0	0	6 (10)	0	0
Cardiovascular AEs						
Cardiac arrhythmias^h^	3 (5)	1 (2)	0	2 (3)	0	0
Heart failure^i^	1 (2)	0	0	3 (5)	1 (2)	1 (2)
Hypotension^j^	1 (2)	0	0	0	0	0
Acute renal failure^k^	2 (4)	0	0	5 (9)	2 (3)	0
New primary malignancy	0	0	0	1 (2)	0	1 (2)

^a^Pooled rate of preferred terms thrombocytopenia and platelet count decreased

^b^Pooled rate of preferred terms neutropenia and neutrophil count decreased

^c^Pooled rate of anemia and red blood cell analyses

^d^Pooled rate of pneumonia, lung infection, and bronchitis

^e^Pooled rate of eight preferred terms in the high-level terms of liver function analyses, tissue enzyme analyses, hepatic enzyme and function abnormalities, protein metabolism disorders, and peritoneal and retroperitoneal disorders; grade 3 events included 1 reversible elevation of alanine aminotransferase in a non-active hepatitis B-carrying patient, 1 hypoalbuminemia reported concurrently with progressive disease (and with no other liver function test abnormalities), and 1 transient increase in blood alkaline phosphatase in the context of development of complete response; no events were classed as serious adverse events or led to discontinuation

^f^Pooled rate of 19 rash-related preferred terms

^g^Modified high-level term of peripheral neuropathies not elsewhere classified

^h^Cardiac arrhythmias standardized MedDRA query (SMQ)

^i^Modified cardiac failure SMQ

^j^Modified vascular hypotensive disorder high-level term and vascular test high-level term

^k^Acute renal failure SMQ

The only SAEs reported in >1 patient on either arm were pneumonia (7 [12%] versus 4 [7%] patients on the ixazomib-Rd versus placebo-Rd arms), lung infection (2 [4%] versus 3 [5%]), and bronchitis, herpes zoster, and hypokalemia (each 2 [4%] versus 0 patients). On-study deaths (occurring within 30 days of last dose of study drug) were reported for 4 (7%) and 5 (9%) patients in the ixazomib-Rd and placebo-Rd arms, respectively. On the ixazomib-Rd arm, 1 patient died from disease progression and 2 patients died from pneumonia, events that were not considered treatment-related, and 1 patient died from treatment-related multiple organ dysfunction syndrome. On the placebo-Rd arm, 3 patients died from disease progression and 1 patient died from pneumonia, events that were not considered treatment-related, and 1 patient died from treatment-related intracranial hemorrhage.

### Pharmacokinetics

Pharmacokinetic assessments demonstrated that ixazomib was rapidly absorbed after weekly oral administration in combination with Rd. The median *T*_max_ was 1 h on day 1 (*n* = 19) and 1.25 h on day 15 (*n* = 19). After single-dose ixazomib administration, the geometric mean (% coefficient of variation (%CV)) for *C*_max_ (*n* = 19) and AUC_0–168_ (*n* = 8) were 71.1 (66) ng/mL and 948 (35) h ng/mL, respectively. The corresponding values on day 15 were 90.4 (53) ng/mL (*n* = 19) and 2428 (42) h ng/mL (*n* = 19), respectively. After day 15 administration, the geometric mean (%CV) *t*_½_ of ixazomib was 170 (25) h (*n* = 16). The geometric mean (%CV) accumulation ratio based on AUC_0–168_ (*n* = 8) was 2.55 (35), which is consistent with the once-weekly dosing regimen and the observed *t*_½_.

## Discussion

The randomized, double-blind, placebo-controlled China Continuation study demonstrated that ixazomib-Rd resulted in superior PFS and OS compared with placebo-Rd in Chinese patients with RRMM after one to three prior lines of therapy. Notably, this is the first randomized study in RRMM demonstrating a significant OS benefit with ixazomib-based therapy. The positive treatment effect for the primary endpoint of PFS supports the findings reported from the global TOURMALINE-MM1 study, in which a significant 35% improvement in PFS was demonstrated [13]. In the China Continuation study, benefits were seen across the secondary efficacy endpoints of ORR, DOR, and TTP, which were also improved with ixazomib-Rd versus placebo-Rd in the global study [13], and findings were consistent with respect to the benefits of renal insufficiency reversal (in patients with creatinine clearance <50 mL/min) and anemia control, and lower rates of renal failure and hypercalcemia (clinical manifestations of MM-related organ dysfunction). Reflecting the global study [13], the China Continuation study showed limited additional toxicity with ixazomib-Rd compared to placebo-Rd. These findings thus provide supportive evidence for the activity, tolerability, and safety of the all-oral triplet regimen of ixazomib-Rd in patients with RRMM. Findings from phase III studies of carfilzomib, elotuzumab, and daratumumab in combination with Rd have similarly demonstrated significant efficacy improvements in patients with RRMM but have not yet reached final OS conclusions, with pomalidomide-based regimens also demonstrating notable activity in this setting [17]. In the context of balancing safety, efficacy, and convenience, ixazomib-Rd represents an additional treatment option for RRMM patients.

Ixazomib-Rd showed consistent benefit over placebo-Rd in both the China Continuation and global TOURMALINE-MM1 studies, with HRs of 0.598 and 0.742 for PFS, and 0.583 and 0.712 for TTP [13], respectively. The studies had identical eligibility criteria, stratification factors, dosing regimens, and efficacy assessment methodologies; however, despite this, median PFS and TTP and response rates in the China Continuation study were lower in both treatment arms than in the global study. These differences are likely attributable to the fact that Chinese MM patients present with more advanced-stage disease, renal dysfunction, and bone destruction than patients in other regions [4, 7]; consequently, there were differences in some baseline characteristics associated with prognosis between the China Continuation and global TOURMALINE-MM1 study populations. For example, Chinese patients had a higher rate (37 vs 22% [Millennium Pharmaceuticals, Inc., data on file]) of ISS stage III disease at initial diagnosis and showed a shorter median time from initial diagnosis to study entry (28.7 and 42.8 months, respectively), indicating more aggressive disease. Furthermore, Chinese patients had more adverse disease status at study entry than the global population [13] (53 vs 11% refractory; 23 vs 12% relapsed and refractory; 24 vs 77% relapsed) and were more heavily pretreated (56 vs 39% had received two or three prior therapies; 84 vs 45% had prior thalidomide exposure; 63 vs 12% were thalidomide-refractory at study entry), but prior stem cell transplant was substantially less common (17 vs 57%) [13]. The presence of high-risk cytogenetic factors is also established as an important prognostic factor in MM that might have affected outcomes [18]; however, as described in the “Statistical analysis” section of the “Methods” section, a limitation of this study was that cytogenetics information was not available for the majority of patients and is thus not reported.

Of note, there was also a relevant difference in median PFS between the MM-021 study of Rd in Chinese patients with RRMM, in which median PFS was 8.3 months [14], and global studies of Rd, in which median PFS was 15–18 months [13, 19–21]. PFS among patients in the placebo-Rd arm of the China Continuation study appeared somewhat shorter than in the MM-021 study of Rd in Chinese patients with RRMM [14]; acknowledging the limitations associated with cross-trial comparisons, this discrepancy may have been due to patient population differences and the use of blinded IRC versus investigator assessment of progression.

Importantly, due to the more aggressive nature of MM in Chinese patients, OS data were mature after a shorter follow-up period in the China Continuation versus the global TOURMALINE-MM1 study, in which median OS had not been reached in either arm after a median follow-up of 23 months. Prolonged follow-up is required to elucidate OS benefit in the global study [13], whereas these data from the China Continuation study provide an earlier demonstration of the OS benefit with ixazomib-Rd versus placebo-Rd in a patient population with more advanced disease. At the final analysis for OS, after a median follow-up of ~20 months, ixazomib-Rd resulted in a highly significant improvement in OS versus placebo-Rd in Chinese patients with RRMM. Additional analyses suggest that these OS findings are indicative of a treatment effect, rather than an effect driven by imbalances between arms in patient subgroups, subsequent therapies, or non-MM-related deaths. The OS benefit was maintained regardless of whether patients received subsequent therapy, and subsequent therapies were similar between arms as a consequence of the double-blind study design (i.e., no imbalance in use of rescue proteasome inhibition), except for alkylators and other therapies; an OS benefit with ixazomib-Rd was observed in patients both with and without subsequent alkylator use, indicating that asymmetry in subsequent alkylator use was not driving the OS benefit. Notably, outcomes in Chinese patients are not confounded by the broad array of approved and investigational regimens available in North America and Europe, and consequently, subsequent therapy in China is different from in Western populations; this supports the demonstrated OS benefit as a true treatment effect of ixazomib-Rd. Interestingly, OS from the start of subsequent bortezomib was similar in both treatment groups, suggesting that bortezomib-based regimens can be given after ixazomib-based treatment. Finally, the proportion of deaths with myeloma as the primary cause was similar in both arms, indicating no imbalance in other-cause mortality driving the OS benefit. However, it is important to acknowledge that caution is required when interpreting the findings from the present study, including the OS data. A limitation of the study, particularly when interpreting OS in patient subgroups, is the relatively low sample size and the small absolute numbers of events.

Tolerability and safety data from the China Continuation study reinforced findings from the global study showing that ixazomib adds limited toxicity to Rd [13]. In Chinese patients, rates of grade ≥3 AEs were similar between arms. Thrombocytopenia was more common in the ixazomib arm, likely associated with the known transient and cyclical decreases in platelet count reported with proteasome inhibition [22, 23], and rates of low-grade gastrointestinal toxicities were also higher. The overall rate of herpes zoster with ixazomib-Rd was 21% but only 9% in patients receiving appropriate antiviral prophylaxis, demonstrating the importance of this concomitant medication in this population with advanced disease. Rates of rash and peripheral neuropathy were similar between arms in the China Continuation study, despite being somewhat higher with ixazomib-Rd in the global study [13]. There were no cardiac or renal safety signals with ixazomib-Rd in Chinese patients. The differences in relative safety profiles between the China Continuation and global studies may be influenced by differences in AE reporting practices between Chinese and global physicians or differences in treatment exposure; patients had received a median of 9 and 6.5 cycles of ixazomib-Rd and placebo-Rd, respectively, at the final analysis for OS in the China Continuation study, compared with medians of 17 and 15 cycles in the global study [13]. However, this shorter treatment duration was not due to poorer tolerability; the rate of discontinuations due to AEs was only 9 and 10% with ixazomib-Rd and placebo-Rd in Chinese patients, respectively, compared with 17 and 14%, respectively, in the global study [13].

**Fig. 4:**
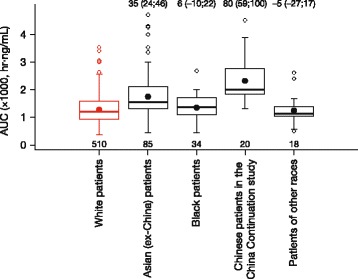
Summary of individual predicted ixazomib systemic exposure for patients receiving ixazomib 4.0 mg. Ixazomib systemic exposures (AUC) were calculated for individual patients in the China Continuation study who underwent pharmacokinetic sampling, as well as for individual patients enrolled in other ixazomib studies (including the TOURMALINE-MM1 study), using the previously reported population pharmacokinetic model [26]. *Red-* and *black*-*filled circles* indicate the mean exposure in White patients and in other race categories, respectively. *Numbers* (*brackets*) *at the top of the plot* show the percent change in mean AUC (with 95% confidence intervals) in other race categories relative to White patients. *Numbers at the bottom of the plot* show the number of patients in each category

Similar to what has been observed in other studies [24, 25], ixazomib was rapidly absorbed in Chinese patients (median *T*_max_ of ~1–2 h post-dose) and exhibited a long *t*_½_ of ~1 week. However, systemic exposures of ixazomib in Chinese patients in this study were higher than exposures observed in other patient populations. Specifically, analysis of pharmacokinetic data from this study using the previously reported population pharmacokinetic model [26] demonstrated that mean ixazomib AUC in Chinese patients was 80% higher than in White patients; however, some overlap in the AUC distribution was observed across patient populations (Fig. 4). The reason for the higher systemic exposures in Chinese patients is unclear. Differences in body size are known to exist between Asian and Western patient populations. Based on population pharmacokinetic analyses [26, 27], neither weight (range 37–151 kg) nor body surface area (range 1.2–2.7 m^2^) was a significant covariate on ixazomib clearance, suggesting that the higher ixazomib exposures observed in Chinese patients are not related to differences in body size. Inter-ethnic differences in the expression and activity of cytochrome P450 (CYP) enzymes or drug transporters have also been reported between Asian and non-Asian patient populations [28, 29]; examples include differences in allelic frequencies for CYP2C19, and lower intrinsic activity of the hepatic uptake transporter OATP1B1 in Asian populations compared with non-Asian populations [30, 31]. However, at clinically relevant ixazomib concentrations, no specific CYP enzyme predominantly contributes to ixazomib metabolism and ixazomib is not a substrate for hepatic organic anion-transporting polypeptides [11]. Therefore, inter-ethnic differences in drug-metabolizing enzymes or transporters are unlikely to explain the higher ixazomib exposures observed in this study. Importantly, although ixazomib systemic exposures were higher in Chinese patients, the 4.0 mg weekly ixazomib dose, in combination with Rd, demonstrated a favorable benefit–risk profile in Chinese patients with RRMM.

## Conclusion

The China Continuation study of ixazomib-Rd versus placebo-Rd demonstrated consistent and significant superiority with ixazomib-Rd for the primary endpoint of PFS, with limited additional toxicity. Further, the China Continuation study showed a significant improvement in OS with ixazomib-Rd at the final analysis. These findings support the efficacy and tolerability of this all-oral triplet regimen as a treatment option for patients with RRMM around the world.

## Additional files

Additional file 1: Supplementary methods. Hou C16010 China Continuation study J Hematol Oncol. (DOCX 38 kb)

Additional file 2: Supplementary results. Hou C16010 China Continuation study J Hematol Oncol. (DOCX 40 kb)

Additional file 3: Details of all sites’ ethics committee approvals. (DOC 44 kb)

## Abbreviations

%CV: % coefficient of variation
AE: Adverse event
CI: Confidence interval
CYP: Cytochrome P450
DOR: Duration of response
ECOG: Eastern Cooperative Oncology Group
HR: Hazard ratio
IRC: Independent review committee
ISS: International Staging System
MM: Multiple myeloma
OR: Odds ratio
ORR: Overall response rate
OS: Overall survival
PD: Progressive disease
PFS: Progression-free survival
PR: Partial response
Rd: Lenalidomide–dexamethasone
RRMM: Relapsed/refractory multiple myeloma
SAE: Serious adverse event
SD: Stable disease
TTP: Time to progression
VGPR: Very good partial response
