# Supplementary material for: Randomized, double-blind, placebo-controlled phase III study of ixazomib plus lenalidomide-dexamethasone in patients with relapsed/refractory multiple myeloma: China Continuation study
Source: J Hematol Oncol. 2017 Jul 6;10:137. doi: 10.1186/s13045-017-0501-4 (PMC5500972; doi:10.1186/s13045-017-0501-4)
Supplement: Supplementary file 1 — Supplementary methods. Hou C16010 China Continuation study J Hematol Oncol. (DOCX 38 kb) [file 13045_2017_501_MOESM1_ESM.docx]

Randomized, double-blind, placebo-controlled phase III study of ixazomib plus lenalidomide-dexamethasone in patients with relapsed/refractory multiple myeloma: China Continuation study

Jian Hou,^1^ Jie Jin,^2^ Yan Xu,^3^ Depei Wu,^4^ Xiaoyan Ke,^5^ Zhou Daobin,^6^ Jin Lu,^7^ Xin Du,^8^ Xiequn Chen,^9^ Junmin Li,^10^ Jing Liu,^11^ Neeraj Gupta,^12^ Michael J. Hanley,^12^ Hongmei Li,^12^ Zhaowei Hua,^12^ Bingxia Wang,^12^ Xiaoquan Zhang,^12^ Hui Wang,^12^ Helgi van de Velde,^12^ Paul G. Richardson,^13^ Philippe Moreau^14^

# SUPPLEMENTARY METHODS

## Eligibility criteria

The China Continuation study was a separate regional expansion of the global phase III TOURMALINE-MM1 study and had identical eligibility criteria. Briefly, patients aged ≥18 years with a confirmed diagnosis of MM, who had measurable disease (by serum or urine protein electrophoresis, and/or free light chain [FLC] assay) and creatinine clearance ≥30 mL/min, and who had relapsed and/or refractory disease having received one to three prior treatments, were eligible. Patients who had received prior bortezomib and/or prior immunomodulatory drugs were eligible, including patients who were refractory to prior thalidomide-based treatment, as were patients who had relapsed/refractory disease or who were refractory to all prior therapies. Patients refractory to previous bortezomib-based or lenalidomide-based treatment were excluded. Refractory disease was defined as progression on treatment or within 60 days after the last dose of therapy.

## Pharmacokinetics analyses

The pharmacokinetics of ixazomib were characterized in a subset of Chinese patients who were randomized to ixazomib-Rd and who consented to intensive pharmacokinetic sampling. Blood samples for measurement of ixazomib plasma concentrations were collected from consenting patients on both study arms at multiple time points following the day 1 (0 to 168 h post-dose) and day 15 (0 to 336 h post-dose) ixazomib doses in cycle 1. Plasma concentrations of ixazomib were measured using a validated liquid chromatography/tandem mass spectrometry assay (Gupta N, Hanley MJ, Venkatakrishnan K, et al. The effect of a high-fat meal on the pharmacokinetics of ixazomib, an oral proteasome inhibitor, in patients with advanced solid tumors or lymphoma. *J Clin Pharmacol*. 2016;56:1288–95). After unblinding, pharmacokinetic parameters were estimated using non-compartmental analysis methods using Phoenix WinNonlin version 6.2 (Pharsight, Princeton, NJ). Population pharmacokinetic data analysis was performed using NONMEM software (version 7.2) for non-linear mixed effects models, running under Perl-speaks-NONMEM (PsN 4.2.0) on a grid of CentOS 7.0 Linux servers, and the Intel Fortran compiler, version 12.

## Statistical analysis – events required for final analyses of PFS and OS

Patients enrolled in the China Continuation study were not included in the global TOURMALINE-MM1 intent-to-treat population, and data for the China Continuation study were analyzed based on a separate statistical analysis plan. The sample size of 115 patients was intended to fulfill Chinese regulatory requirements with the goal of evaluating consistency with the global study in the treatment effect of ixazomib-Rd versus placebo-Rd. There was no formal power calculation for any of the outcomes, and the sample size was not determined based upon the findings from the global TOURMALINE-MM1 study or an attempt to demonstrate non-inferior or equivalent efficacy benefit in the China Continuation study. However, simulations were conducted at the study design stage to determine the power to observe a specific effect size for the primary endpoint based on the specified sample size.

A final analysis of PFS was to be conducted either 18 months after the first patient enrolled in the China Continuation study or when a total of 60 PFS events were reached for patients in China, whichever came first. With HR = 0.728, the probability of observing HR <0.9 is estimated to be ~80% with ~60 PFS events. At a data cut-off of July 12, 2015, the target number of PFS events had been observed; PFS and response data presented herein are based on this final analysis of PFS.

The study continued beyond the final analysis for PFS in a double-blind placebo-controlled fashion to monitor for OS and AEs; a final analysis for OS was to be conducted either 24 months after the last patient enrolled in the China Continuation study or when a total of 60 deaths – or 50% of patients with death events – had been reported for patients in China, whichever came first. At a data cut-off of July 19, 2016, the required number of deaths had been observed. OS, treatment exposure, and safety data are reported from this final analysis for OS.
