# Supplementary material for: Randomized, double-blind, placebo-controlled phase III study of ixazomib plus lenalidomide-dexamethasone in patients with relapsed/refractory multiple myeloma: China Continuation study
Source: J Hematol Oncol. 2017 Jul 6;10:137. doi: 10.1186/s13045-017-0501-4 (PMC5500972; doi:10.1186/s13045-017-0501-4)
Supplement: Supplementary file 2 — Supplementary results. Hou C16010 China Continuation study J Hematol Oncol. (DOCX 40 kb) [file 13045_2017_501_MOESM2_ESM.docx]

Randomized, double-blind, placebo-controlled phase III study of ixazomib plus lenalidomide-dexamethasone in patients with relapsed/refractory multiple myeloma: China Continuation study

Jian Hou,^1^ Jie Jin,^2^ Yan Xu,^3^ Depei Wu,^4^ Xiaoyan Ke,^5^ Zhou Daobin,^6^ Jin Lu,^7^ Xin Du,^8^ Xiequn Chen,^9^ Junmin Li,^10^ Jing Liu,^11^ Neeraj Gupta,^12^ Michael J. Hanley,^12^ Hongmei Li,^12^ Zhaowei Hua,^12^ Bingxia Wang,^12^ Xiaoquan Zhang,^12^ Hui Wang,^12^ Helgi van de Velde,^12^ Paul G. Richardson,^13^ Philippe Moreau^14^

# SUPPLEMENTARY RESULTS

## Sensitivity analyses of overall survival

To adjust for potential confounding effects of subsequent therapies after patients discontinued study treatment, two sensitivity analyses of OS were conducted using marginal structural models (MSM) and inverse probability of censoring weight (IPCW) Cox models. Both approaches took two steps in the analysis. In the first step, pooled logistic regression models (PLRMs) were used to compute probability leading to receiving subsequent therapies (and censoring in the MSM). Baseline and time-variant covariates that were statistically significant in at least one of the PLRMs or clinically associated with receiving subsequent therapies or patient prognosis were included in PLRMs. After testing, the following baseline or post-baseline covariates were included in the final PLRMs: hemoglobin, urine, M-protein, serum M-protein, platelets, and Eastern Cooperative Oncology Group performance status score, as well as the study stratification factors. In the second step of the analysis, extended Cox proportional hazard regression models, which included multiple observations per patients, were used for the OS analyses by both the MSM and IPCW approaches. Robust variance was used in the modeling to take into account the intra-patient correlation among possible multiple observations per patient over time. The stabilized weights that were derived from the first step were applied for each patient at each observation in the Cox modeling.

Results of the MSM and IPCW analyses are as shown in Table S1, along with the intent-to-treat (ITT) Kaplan–Meier analysis result, which did not adjust for possible confounding due to receiving subsequent therapy. Results from the MSM and IPCW approaches were consistent with those from the ITT Kaplan–Meier analysis and support the ITT findings and conclusions. An acknowledged limitation of the MSM and IPCW analyses is that it is not certain whether all potential confounding factors have been included in the adjusted analyses.

## Table S1. Summary of analysis of overall survival with ixazomib-Rd versus placebo-Rd using different assessment methodologies (ITT population)

| **Method** | **Hazard ratio (95% CI)** | ***p*-value** |
| --- | --- | --- |
| Kaplan–Meier | 0.419 (0.242–0.672) | 0.001 |
| Marginal structural models | 0.256 (0.097–0.672) | 0.0056 |
| Inverse probability of censoring weighted | 0.271 (0.105–0.696) | 0.0067 |

## Table S2. Subsequent therapy received by patients in the ixazomib-Rd or placebo-Rd arm

| **Subsequent therapy** | **Ixazomib-Rd** | Placebo-Rd |
| --- | --- | --- |
| Any, *n*/*N* (% of total population) | 30/57 (53) | 25/58 (43) |
| Types of subsequent therapy, n (% of patients who received subsequent therapy) | *N =* 30 | *N* = 25 |
| Corticosteroids | 26 (87) | 20 (80) |
| Proteasome inhibitors | 15 (50) | 11 (44) |
| Bortezomib | 15 (50) | 11 (44) |
| Immunomodulatory drugs | 17 (57) | 15 (60) |
| Lenalidomide | 1 (3) | 0 |
| Thalidomide | 16 (53) | 15 (60) |
| Alkylators | 24 (80) | 14 (56) |
| Cyclophosphamide | 21 (70) | 11 (44) |
| Melphalan | 8 (27) | 4 (16) |
| Other^a^ | 18 (60) | 11 (44) |

^a^Predominantly cisplatin, clarithromycin, etoposide (9–10% of total population); also, doxorubicin, epirubicin (hydrochloride), ifosfamide, liposomal doxorubicin hydrochloride, pirarubicin, vincristine, vindesine, vinorelbine, and any other antineoplastic agents/therapeutic products (≤3% of total population)
