# Supplementary material for: Randomized, double-blind, placebo-controlled phase III study of ixazomib plus lenalidomide-dexamethasone in patients with relapsed/refractory multiple myeloma: China Continuation study
Source: J Hematol Oncol. 2017 Jul 6;10:137. doi: 10.1186/s13045-017-0501-4 (PMC5500972; doi:10.1186/s13045-017-0501-4)
Supplement: Supplementary file 3 — Details of all sites’ ethics committee approvals. (DOC 44 kb) [file 13045_2017_501_MOESM3_ESM.doc]

| **Site No** | **Site Name** | **Name of IEC** | **Contact Info of IEC** | **Date of Approval** | **Approval File No** |
| --- | --- | --- | --- | --- | --- |
| 09001 | Shanghai Changzheng Hospital | Ethics Committee of Shanghai Changzheng Hospital | Address: No.415, Fengyang Road, Shanghai, PRC. 200003  Tele: (+86) 21 8188 6191  Email: [Haijunmiao@126.com](mailto:Haijunmiao@126.com) | 9Mar2014 | 2014(伦审)-07 |
| 09003 | Institute of Hematology & Blood Diseases Hospital, Chinese Academy of Medical Sciences | Ethics Committee of Institute of Hematology & Blood Diseases Hospital, Chinese Academy of Medical Sciences | Address: No.288, Nanjing Road, Heping District, Tianjin, PRC. 300041  Tele: (+86) 22 23909237  Email: bloodec@126.com | 18Feb2014 | XY2014001-EC-1 |
| 09004 | Peking Union Medical College Hospital | Ethics Committee of Peking Union Medical College Hospital | Address: No.1 Shuaifuyuan, Wangfujing, Dongcheng Distric, Beijing, PRC. 100006  Tele: (+86) 10 6915 8355  Email: [cprc@pumch.cn](mailto:cprc@pumch.cn) | 18Jun2014 | KS2014048 |
| 09005 | The First Affiliated Hospital, College of Medicine, Zhejiang University | Ethics Committee of the First Affiliated Hospital, College of Medicine, Zhejiang University | Address: No.79 Qingchun Road, Hangzhou 310003 Zhejiang  Tele: (+86) 571 87236685  Email: [yixuelunli123@163.com](mailto:yixuelunli123@163.com) | 21Jan2014 | 2013伦审第(100) 号 |
| 09006 | Peking University Third Hospital | Peking University Third Hospital Medical Science Research Ethics Committee | Address: No.49 Huayuanbei Road, Haidian District, Beijing PRC 100089  Tele: (+86) 10 8226 5571  Email: [nxy178037@163.com](mailto:nxy178037@163.com) | 6Jan2014 | (2014) 药伦审第(04)号 |
| 09007 | Peking University Peoples’ Hospital | Peking University Peoples’Hospital Medical Ethics Committee | Address: No.11 Xizhimen South Street Xicheng District, Beijing PRC 100032  Tele: (+86) 10 8832 4516  Email:rmyyyljd@163.com | 15Apr2014 | (2014) 伦审药临字第(06)号 |
| 09008 | Xiangya Third Hospital of Central South University | Ethics Committee of Xiangya Third Hospital of Central South University | Address: Tongzipo Road, Yuelu District, Changsha Hunan, PRC 410013  Tele: (+86) 731 8861 8938  Email:xy3irb@163.com | 14Mar2014 | 14015 |
| 09009 | First Affiliated Hospital of Fourth Military Medical University | Independent Ethics Committee of First Affiliated Hospital of Fourth Military Medical University | Address: No.127 Changle Road, Xi’an Shanxi, PRC 710032  Tele: (+86) 29 8477 1794  Email:xjyyllwyh@163.com | 21Feb2014 | 第20140115-3号 |
| 09010 | The 1st Affiliated Hospital of Soochow University | Ethics Committee of the 1st Affiliated Hospital of Soochow University | Address: No.188 Shizi Street, Suzhou Jiangsu, PRC 215006  Tele: (+86) 512 6778 0081  Email:sdfyec@163.com | 20Jan2014 | (2014) 伦药批第(21)号 |
| 09011 | Guangdong People Hospital | Ethics Committee of Guangdong People Hospital | Address: No.106 Zhongshan 2^nd^ Road, Guangzhuo Guangdong, PRC 510080  Tele: (+86) 20 8382 7812 ext 20870  Email:gdghospital_ec@163.com | 5Mar2014 | 粤医伦理(2014) 9号 |
| 09013 | Shanghai Ruijin Hospital, Affiliated Hospital of Shanghai Jiaotong University Medical College | Ethics Committee of Ruijin Hospital | Address: No.197 Ruijin 2^nd^ Road, Shanghai, PRC 200025  Tele: (+86) 21 6437 0045 ext 665824  Email: wyfkjc@163.com | 11Jun2014 | (2014) 伦审第(37)号 |
